# Nitrogen and Ergosterol Concentrations Varied in Live Jack Pine Phloem Following Inoculations With Fungal Associates of Mountain Pine Beetle

**DOI:** 10.3389/fmicb.2020.01703

**Published:** 2020-07-22

**Authors:** Sydne Guevara-Rozo, Altaf Hussain, Jonathan A. Cale, Jennifer G. Klutsch, Rahmatollah Rajabzadeh, Nadir Erbilgin

**Affiliations:** Department of Renewable Resources, University of Alberta, Edmonton, AB, Canada

**Keywords:** *Dendroctonus ponderosae*, fungal biomass, microbial symbionts, ophiostomatoid fungi, *Pinus banksiana*

## Abstract

Bark beetles form symbiotic associations with multiple species of fungi that supplement their metabolic needs. However, the relative contributions of each symbiont to the nutrition of bark beetles have been largely unexplored. Thus, we evaluated the ability of three fungal symbionts of mountain pine beetle to concentrate nitrogen and produce ergosterol while infecting phloem of a novel host jack pine. Ergosterol was used as proxy to determine the fungal biomass (hyphal density) in the current study. We inoculated 80 trees in two forest stands with one of the three fungal species or a non-fungal (control) agar. Six weeks later, we collected phloem from the necrotic lesions induced by the fungi, uninfected tissues adjacent to lesions, and non-inoculated control trees. We found that nutritional contributions varied with fungal species. Nitrogen in lesions was higher in trees inoculated with *Ophiostoma montium* or control trees, relative to *Grosmannia clavigera* or *Leptographium longiclavatum*. Furthermore, concentrations of ergosterol were higher in *O. montium* lesions compared to other tissues or treatments. These results suggest that *O. montium* differs from *G. clavigera* and *L. longiclavatum* in terms of acquiring nitrogen from host tissues and producing ergosterol.

## Introduction

Bark beetles (Coleoptera: Curculionidae, Scolytinae) contain some of the most ecologically and economically important insect species of conifer forests due to their ability to kill a large number of trees during periodic outbreaks, with cascading consequences for ecosystem function (Kurz et al., 2008; Bentz et al., 2010; Hicke et al., 2012; Seidl, 2014). Fungal symbionts associated with bark beetles can amplify the beetles’ damage by promoting successful host colonization and development (Harrington, 2005; DiGuistini et al., 2007; Six and Wingfield, 2011; Six, 2012; Wang et al., 2013, 2014; Therrien et al., 2015; Cale et al., 2017; Zhao et al., 2019). In particular, fungal symbionts can facilitate beetle nutrition by either serving as a direct dietary substrate (e.g., ergosterol), or indirectly concentrating host plant nutrients (e.g., nitrogen) (Ayres et al., 2000; Bleiker and Six, 2007; Goodsman et al., 2012). Although bark beetle species have multiple species of symbiotic fungi (Klepzig and Six, 2004; Klepzig et al., 2004; Roe et al., 2011a), how these fungi differ in the nutritional benefits they provide to the beetles is poorly understood.

Mountain pine beetle (*Dendroctonus ponderosae* Hopkins; MPB) has killed millions of hectares of pine (*Pinus*) forests over the past two decades in western North America (Bentz et al., 2010; Safranyik et al., 2010). In Canada, MPB outbreaks were historically restricted to lodgepole pine (*Pinus contorta* Douglas ex Loudon var. *latifolia* Engelm. ex S. Watson) forests; however, the beetle has recently expanded into jack pine (*Pinus banksiana* Lamb) forests in British Columbia and southern Alberta and threatens to move toward eastern boreal forests (Cullingham et al., 2011; Erbilgin, 2019). The life cycle of MPB is well described (Safranyik et al., 2010). Briefly, the host tree colonization begins with the arrival of a female beetle at a potential new host. Female beetles initiate the mass aggregation on the hosts by attracting both sexes of beetles. After successful host colonization and mating, female beetles lay eggs under the bark (phloem) and inoculate tree phloem with symbiotic ophiostomatoid fungi (Ascomycota: Ophiostomataceae). These fungi help beetles to overcome host defenses by infecting host vascular tissues and disrupting xylem function. Fungi sporulate in beetle galleries and following hatching, larvae feed exclusively on the phloem and hyphae of the inoculated fungi through their four instars of development. Once pupation occurs, immature adults primarily feed on the fungal spores prior to emergence from the host. Beetles emerging from the natal host carry fungal spores in a specialized structure called the mycangia or phoretically on their exoskeleton.

The three most common fungal associates of MPB are *Grosmannia clavigera* (Robinson-Jeffery and Davidson) Zipfel, de Beer, and Wing., *Ophiostoma montium* (Rumford) von Arx, and *Leptographium longiclavatum* Lee, Kim and Breuil (Lee et al., 2005, 2006a). Abundance and diversity of fungi on beetles show spatial and temporal variation across the range of MPB (Six and Bentz, 2007; Roe et al., 2011a). These fungi commonly co-exist and environmental conditions can affect their relative abundance (Six and Bentz, 2007; Bleiker et al., 2009; Roe et al., 2011b; Ojeda-Alayon et al., 2017). *Grosmannia clavigera* and *L*. *longiclavatum* appear to be more cold tolerant than *O. montium* and therefore latitude strongly influences their relative abundance, with *L. longiclavatum* being most abundant at more northern latitudes (Kenkel et al., 1997; Six and Bentz, 2007; Roe et al., 2011a, b; Ojeda-Alayon et al., 2017).

Bark beetle development requires nutrients that either have low availability or are absent in the host tissues. The nitrogen content of tree phloem is much lower than that required by bark beetles (Mattson, 1980). It is generally thought that symbiotic fungi supplement MPBs’ low nitrogen diet by concentrating it within fungal hyphae and conidia (asexual spores) surrounding larvae in the phloem (Ayres et al., 2000; Bleiker and Six, 2007; Cook et al., 2010; Goodsman et al., 2012). Furthermore, sterols are required by insects in production of structurally and functionally different lipids and also act as precursors of hormones that regulate insect development (Clayton, 1964; Behmer and Nes, 2003). However, bark beetles must acquire them through their diet including their fungal symbionts (Behmer and Nes, 2003).

For many ophiostomatoid fungi, such as *G. clavigera, O. montium*, and *L. longiclavatum*, the dominant sterol in cells is ergosterol (C_28_H_44_O) (Behmer and Nes, 2003). Ergosterol is not produced by plants or animals but is a critical component of fungal cell membranes and acts to maintain cell membrane integrity and fluidity which has been shown to be essential for aerobic growth of most fungi. Ergosterol is required by beetles for larval development, pupation, and egg viability (Chu et al., 1970; Morales-Ramos et al., 2000; Behmer and Nes, 2003). For instance, ergosterol is the sole sterol that allows the complete development of several beetle species; in fact, the absence of the fungal associates, and thus ergosterol, increased their mortality and interrupted pupation (Chu et al., 1970; Kok et al., 1970; Morales-Ramos et al., 2000). Similarly, the fungi associated with MPB are essential for the successful development of the beetles (Six and Paine, 1998) as the ergosterol provided by these symbionts is likely the primary sterol source for MPB larvae (Behmer and Nes, 2003; Bentz and Six, 2006). In this study, we investigated nitrogen and ergosterol concentrations of all three fungal species, *G. clavigera, O. montium*, and *L. longiclavatum*. Ergosterol was used as proxy to determine the fungal biomass (hyphal density) in the current study. Determining the nutritional contributions of each fungal species may improve our understanding of why some fungi are more abundant and how they may affect beetles’ host colonization and range expansion.

Currently Bentz and Six (2006) is the only study that has reported ergosterol concentrations associated with the fungal symbionts of MPB. In their study, the authors determined and compared the relative amounts of ergosterol present in phloem tissues surrounding MPB larval galleries in lodgepole pine trees. The study found that tissues surrounding MPB larval galleries contained more ergosterol than non-infested tissues. However, to date, there is no information describing the relative concentrations of ergosterol of different fungal species associated with MPB colonizing lodgepole pine or jack pine trees.

Here, we investigated four research questions: Do nitrogen and ergosterol concentrations vary: (1) between fungal inoculated and mock-inoculated (inoculation without any fungal cultures) jack pine trees, (2) between lesion (reaction zones of tissues in response to fungal infection) and non-infected phloem tissues in inoculated jack pine trees, (3) among the three fungal species, and (4) among different heights along jack pine stems. For the first three questions, we compared the concentrations of nitrogen and ergosterol between inoculated and mock-inoculated trees as well as between different types of tissues (infected vs. non-infected phloem) in the inoculated trees. For the fourth question, we evaluated whether the growth and overall nitrogen and ergosterol concentrations from the fungi vary along the length of the tree stem as height has been shown to influence fungal performance in the MPBs historical host lodgepole pine (Goodsman et al., 2013). We hypothesized that nutrient contributions by *G*. *clavigera* and *L*. *longiclavatum* would be similar to each other due to their closer phylogenetic relationship and their wider niche overlap, but their contributions would differ from those provided by *O*. *montium* due to the farther phylogenetic relationships and narrow niche overlap (Six and Paine, 1999; Rice and Langor, 2009).

## Materials and Methods

### Sampling Collection

We selected 40 healthy jack pine trees (i.e., no visual aboveground evidence of biotic or abiotic stress) from each of two forest stands located near Lac la Biche (55°03′27.9″N 112°01′36.4″W; 55°04′31.7″N 111°59′42.7″W), Alberta (Canada) in early July 2017. Jack pine trees were identified based on their species-specific morphological characteristics as well as monoterpene profiles (Lusebrink et al., 2016). In each stand, we randomly assigned trees (mean diameter at breast height = 21.58 cm ± 0.23 S.E.) to one of four inoculation treatments, with 10 trees per treatment. Treatments consisted of wound inoculations with culture plugs (a plug of 1 cm diameter) of one of three fungi, *G*. *clavigera*, *L*. *longiclavatum*, or *O*. *montium*, or media without any fungal culture as mock inoculations (“mock-inoculated control” hereafter). We inoculated all trees at three heights (1, 3, and 5 m) along the tree bole in the four cardinal directions (N, S, W, E) (one plug in each cardinal direction per height), for a total of 12 inoculations per tree. We used a single isolate of each of *O. montium* (Erbilgin Lab (EL) 005), *G*. *clavigera* (EL033), and *L. longiclavatum* (EL0380) for the inoculations. These fungi were part of our master cultures and were originally isolated from blue-stained sapwood between MPB galleries in *P*. *contorta* (for *O*. *montium*), *P. banksiana* (for *L. longiclavatum*) and *P*. *contorta-P*. *banksiana* hybrid (for *G*. *clavigera*). Details regarding these cultures are provided in Table 1. Master cultures were grown on potato dextrose agar (PDA) at 22 °C in total darkness for 10 days before field inoculations and stored in the Erbilgin laboratory. In the field, one 1 cm diameter plug was removed from the edge of growing colony with a cork borer and used to inoculate trees. At the time of inoculations, we collected additional phloem samples (1 cm in dia.) from each height (15 cm above between two inoculation points) to secure the amount of tissues required for different chemical analyses planned (See details below). These additional tissue samples were collected between the two inoculations points at the same height. We covered the inoculation and sample collection points with plastic wrap to avoid moisture loss and any secondary infections by other fungal species.

**TABLE 1:** Detailed source information about isolates of Ophiostomatoid fungi used during tree inoculations.

Accession number	Location collection	Date collected	Species	Notes
**EL033**	Banff Alberta (Nadir Erbilgin)	May 2016	*Grosmannia clavigera*	Isolated from a MPB gallery in *Pinus contorta* by Nadir Erbilgin
**EL038**	Graham fire base Alberta (Nadir Erbilgin)	May 2016	*Leptographium longiclavatum*	Isolated from a MPB gallery in *Pinus banksiana* x *P. contorta* hybrid by Nadir Erbilgin
**EL005**	Westcastle Alberta (Nadir Erbilgin)	January 2015	*Ophiostoma montium*	Isolated from a MPB gallery in *P. contorta* by Nadir Erbilgin

*All fungi were isolated from mountain pine beetle (MPB) (Dendcroctonus ponderosae) oviposition galleries.*

**FIGURE 1:**
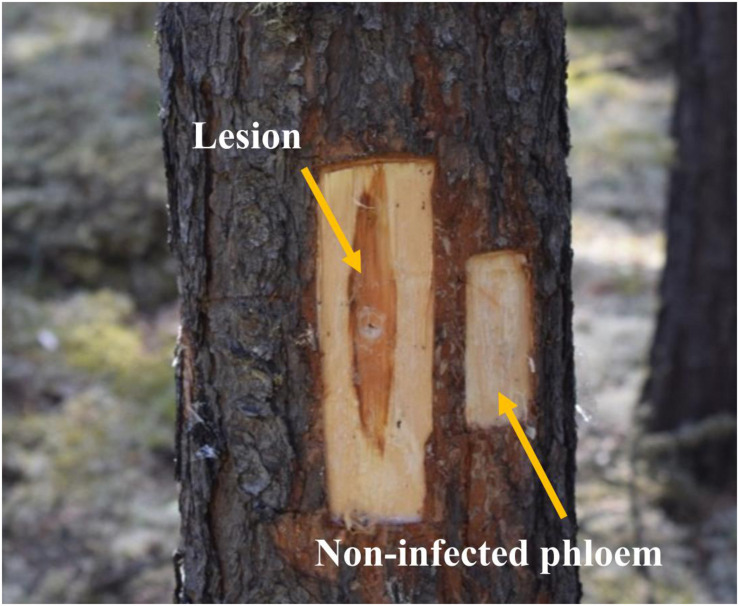
Lesion and non-infected phloem samples collected from jack pine (*Pinus banksiana*) trees. Lesions corresponded to the dark reaction zones on phloem, where typically terpenoid and phenolic compounds are accumulated, in response to the fungal growth. Non-infected phloem corresponded to the non-reaction zones on phloem where fungal growth was not expected.

Six weeks later, we collected phloem tissue samples from lesions as well as non-infected phloem adjacent to lesions (about 4 cm above the lesions; “non-infected phloem” hereafter; Figure 1). During our sampling we observed that fungal inoculations created much longer lesions than mock inoculations, suggesting that our inoculations were successful (Bonello et al., 2006; Cale et al., 2017, 2019). In addition, we confirmed fungal species by subsampling tissues taken from lesions on each tree. We did not have any contamination issue in any of the subsamples taken. At each height, we collected the whole lesion area in the north aspect of trees inoculated with *G*. *clavigera* and *L*. *longiclavatum*. For trees inoculated with *O. montium*, we collected the whole lesion area in both north and east aspects of trees because the size of lesions formed was too small to provide the amount of tissues required for chemical analyses. On each tree, we photographed the sapwood lesion in the north aspect to estimate the fungal area and length using ImageJ software (Abramoff et al., 2004). Similarly, we excised phloem tissue (5 cm × 4 cm) adjacent to the lesions from all fungal inoculated trees (“non-infected phloem” hereafter, Figure 1). From mock-inoculated control trees, we collected one 5 cm x 4 cm phloem sample above the wound inoculation point in the north aspect of trees.

We stored all tissue samples in liquid nitrogen in the field and transferred them to −40°C freezer in the laboratory. For each tree, we pooled samples collected from the four cardinal directions by tissue types and tree height at the time of inoculations (constitutive) and after inoculations (induced). At the time of inoculations, we had two types of tissues: those collected from trees selected for fungal inoculations and those for mock inoculations. After inoculations, tissues were separated as lesions and non-infected phloem, both collected from fungal-inoculated trees, as well as those collected from mock-inoculated control trees. We ground all tissue samples in liquid nitrogen using a mortar and pestle to determine ergosterol and nitrogen concentrations for each fungal species as well as for non-infected phloem and mock-inoculated control trees. In the case of trees inoculated with *O*. *montium*, we pooled lesion samples from the north and east aspects together prior to grinding.

### Nitrogen Analysis

We dried ground tissue (40 mg) from samples collected at 40 °C for 24 h in an oven and transported them to the Natural Resources Analytical Laboratory (NRAL) at the University of Alberta^1^ for total nitrogen determination. Samples were analyzed in a FLASH 2000 HT Organic Elemental Analyzer (Thermo Fisher Scientific Inc., Bremen, GER). We reported nitrogen as a percentage of sample dry weight.

### Ergosterol Analysis

We freeze-dried the ground tissues from lesions, non-infected phloem, and mock-inoculated control trees for four days (Labconco Corp., Kansas City, MO, United States). We extracted ergosterol from 300 mg of the dried sample by suspending it in 5 mL 10% w/v KOH in methanol following the protocol described by Sterkenburg et al. (2015) with slight modifications. Briefly, after 15 min of sonication and 90 min of incubation in a water bath (70°C), we added 1 mL of water to increase the polarity of the methanol phase. After adding 3 mL of pentane, we vortexed samples for 1 min at 3,000 rpm, followed by 5 min centrifugation at 1,000 rpm. We transferred the non-polar phase (upper layer) into a new 20 mL glass tube. We then added 2 mL of pentane to the original tubes and repeated the centrifugation and transfer steps and then pooled resulting extracts. We evaporated pentane from each tube on a 40 °C heating block under N_2_ gas flow until dry and resuspended the samples in 300 μL of hexane containing 0.001 mg/mL of methyl-tricosanoate (internal standard). We heated samples in a water bath (40 °C) for 5 min and sonicated for 3 min before filtering with a glass Pasteur pipette fitted with a plug of glass wool at the tapered end. We injected 2 μL of the extracts into a GC-MS (7890A-5975C, Agilent Tech., Santa Clara, CA, United States) equipped with an HP-5 column (ID 0.320 mm; Film 0.25 μm; length 30 m; Agilent Tech.) using pulsed splitless mode. Helium was used as a carrier gas flowing at 1.5 mL min^–1^, with a temperature program of 50°C for 2 min, then 25°C min^–1^ to 240°C, and then 15°C min^–1^ to 300°C (held for 7 min). We detected and quantified ergosterol and internal standards using scan and selective ion monitoring modes, respectively.

Peak identification and confirmation were based on the retention times of commercial standards of ergosterol (>95% chemical purity, Sigma-Aldrich, ON, CAN) and methyl-tricosanoate (internal standard used in the quantitative determination of ergosterol) (>99% purity, Nu-Chek Prep Inc., MN, United States). We used fragment ions to quantify ergosterol (m/z 363 and 396) and the internal standard (m/z 368). Furthermore, quantifications used a calibration curve with dilutions of the ergosterol standard in hexane at five dilutions that ranged from 2 to 200 μg/mL. We reported ergosterol as μg g^–1^ dry weight of sample.

### Data Analyses

We analyzed the fungal growth (lesion area and length) and total nitrogen concentrations before and after fungal inoculations using a generalized linear mixed model. We used site and tree as random factors, and height and fungal treatments as fixed factors. We used square-root or natural-log transformations of the data to meet model assumptions of normality and homoscedasticity, and visually assessed them using quantile–quantile normality and residual plots. We determined the significance of the fixed effects in the model using Wald chi-square tests. Significant models were followed by *post-hoc* Tukey’s honestly significance difference (HSD) tests.

To test proportions of ergosterol incidence among different types of tissues (lesions, non-infected phloem, and mock-inoculated control trees), we used two-proportion *Z*-tests. The statistical significance of the *P* values was assessed through the Holm’s adjustment method for an experiment-wise error rate of 0.05. We compared ergosterol concentrations in lesions among the three fungal species through a generalized linear mixed model as described above. We square-root transformed the data and eliminated zero values in order to meet the normality and homoscedasticity assumptions of the model.

All analyses were conducted in the R Software Environment (version 3.5.0). Mixed models were calculated using functions available in R package “nlme” version 3.1-140 (Pinheiro et al., 2018). Wald chi-squared tests of model parameter significance were performed using functions available in the “car” package version 3.0-2 for R (Fox and Weisberg, 2011). Tukey HSD tests were conducted using functions available in R package “multcomp” version 1.4-10 (Hothorn et al., 2008).

## Results

### Constitutive Total Nitrogen Concentration Along Tree Bole

Percent nitrogen (mean (%) 0.27 ± 0.05 SE) in samples collected from all trees at the time of the inoculations did not vary significantly among the three heights sampled (1, 3, and 5 m) (*X*^2^_(2)_ = 0.56, *P* = 0.757). Thus, we included only the 1 and 5 heights for the remaining chemical analyses and statistical comparisons.

### Lesion Area and Length in Response to Inoculations

**FIGURE 2:**
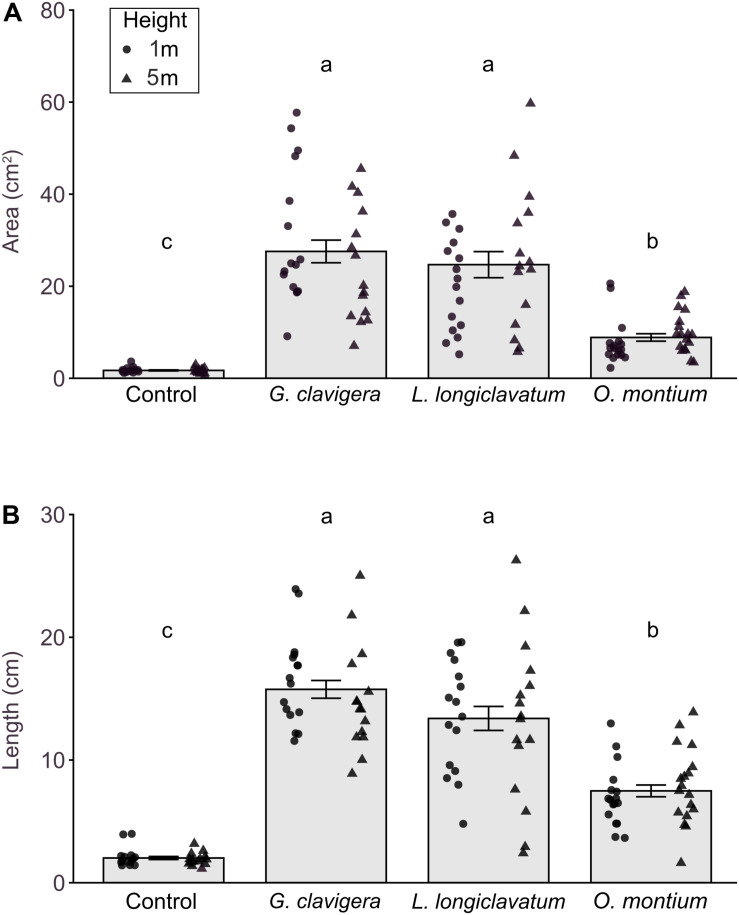
Mean (±SE) lesion areas and lengths in response to inoculations of *Pinus banksiana* with three fungi associated with *Dendroctonus ponderosae*. **(A)** Area and **(B)** length of lesions from trees inoculated with *Ophiostoma montium*, *Grosmannia clavigera*, *Leptographium longiclavatum* fungi, and agar media as a mock control at two heights (1 and 5 m; *n* = 17, 16, 16, and 14 trees, respectively). Samples from different heights were pooled together but the distribution of data was provided for each sample height. Different letters indicate that means are significantly different.

We did not find any significant differences in lesion area (*X*^2^_(1)_ = 0.43, *P* = 0.511) or length (*X*^2^_(1)_ = 0.10, *P* = 0.7485) between 1 m and 5 m tree heights. The interaction effects between fungal treatments and tree heights on lesion area (Figure 2A; *X*^2^_(3)_ = 6.14, *P* = 0.105) or length (Figure 2B; *X*^2^_(3)_ = 1.71, *P* = 0.634) were not significant.

All fungus-inoculated trees had larger lesion areas and longer lengths than did mock-inoculated control trees (Figures 2A,B; *P* < 0.001). Furthermore, lesion area (*X*^2^_(3)_ = 269.91, *P* < 0.001) and length (*X*^2^_(3)_ = 192.40, *P* < 0.001) varied by fungal species, with trees inoculated with *G*. *clavigera* and *L*. *longiclavatum* having larger lesion areas and longer lengths than those inoculated with *O*. *montium*. There was no significant difference in lesion areas and lengths between *G*. *clavigera* and *L. longiclavatum*.

### Total Nitrogen Before and After Inoculations

**FIGURE 3:**
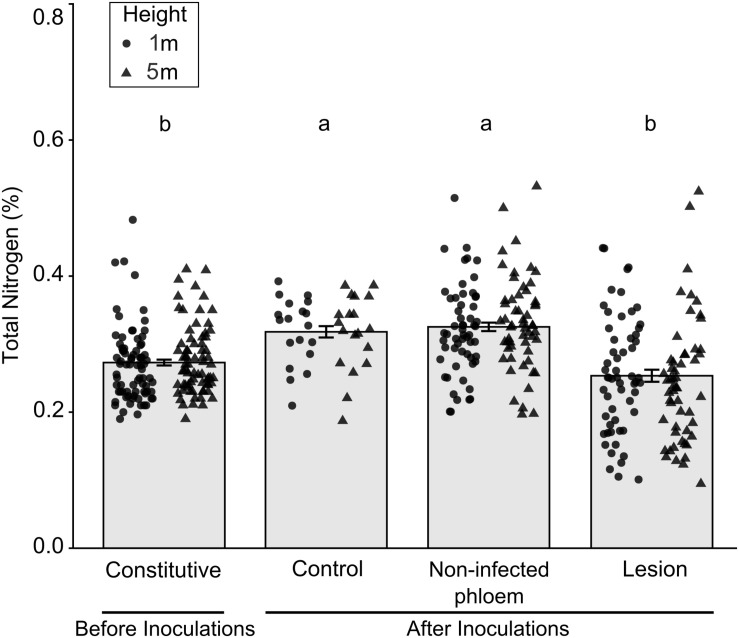
Mean (±SE) total nitrogen in samples collected before and after inoculation of *Pinus banksiana* with three fungi associated with *Dendroctonus ponderosae*. Samples from the different heights were pooled together but the distribution of data was provided for each sample height. Trees were inoculated with *Ophiostoma montium*, *Grosmannia clavigera*, *Leptographium longiclavatum* fungi or mock inoculated (control) at two heights (1 and 5 m). Different letters indicate that means are significantly different.

There were significant differences in percent nitrogen concentrations among tissue types collected before and after inoculations (Figure 3; *X*^2^_(3)_ = 49.46, *P* < 0.001). Total percent nitrogen was higher in tissues from mock-inoculated control trees and non-infected phloem compared to the lesion samples collected before inoculations (constitutive nitrogen). We did not find any differences between tree heights (*X*^2^_(1)_ = 0.72, *P* = 0.396) or tree heights- fungal treatment interactions (*X*^2^_(3)_ = 4.49, *P* = 0.212).

### Total Nitrogen Concentrations Among Fungal Treatments

**FIGURE 4:**
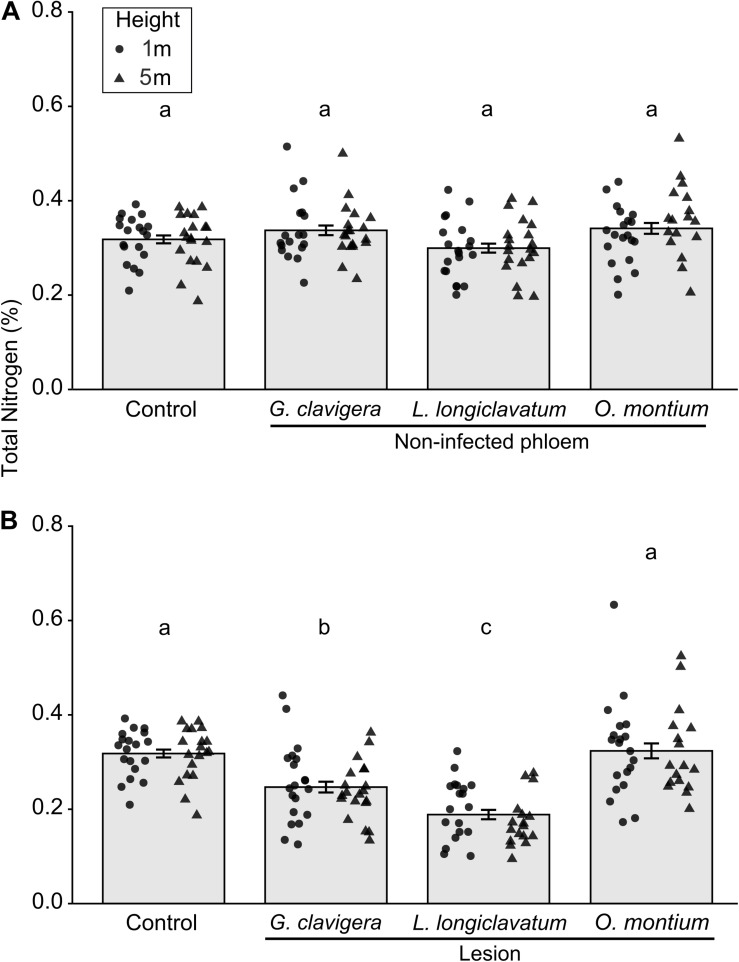
Mean (±SE) total nitrogen from non-infected phloem and lesion tissues and control *Pinus banksiana* trees. **(A)** Percent nitrogen from non-infected phloem of trees inoculated with *Ophiostoma montium*, *Grosmannia clavigera, Leptographium longiclavatum* fungi, or agar media as a control at two heights (1 and 5 m; *n* = 20, 19, 20, and 19 trees, respectively). **(B)** Percent nitrogen among lesions and control. No significant difference was found in total nitrogen between 1 and 5 m stem heights in either non-infected phloem or lesions and samples from the different heights were pooled together but the distribution of data was provided for each sample height. Different letters indicate that means are significantly different among treatments.

Overall, total percent nitrogen was not different between mock-inoculated control trees and non-infected phloem samples (Figure 4A; *X*^2^_(3)_ = 4.78, *P* = 0.195). However, percent nitrogen in non-infected phloem samples did not vary with tree heights (*X*^2^_(1)_ = 0.07, *P* = 0.795) and there was no tree heights-fungal treatment interactions (*X*^2^_(3)_ = 3.00, *P* = 0.391). In contrast, for lesion samples, total percent nitrogen concentrations varied among the four inoculation treatments (Figure 4B; *X*^2^_(3)_ = 42.26, *P* < 0.001). Percent nitrogen in mock-inoculated control trees and *O*. *montium-*induced lesions was higher than that in *G*. *clavigera-* or *L*. *longiclavatum-*induced lesions. Trees inoculated with *L. longiclavatum* had the lowest nitrogen concentrations among the three fungal species. Neither tree heights (*X*^2^_(1)_ = 0.03, *P* = 0.874) nor tree height-fungal treatment interactions were significant (*X*^2^_(3)_ = 1.76, *P* = 0.623) in explaining variation in percent nitrogen in lesion tissue.

### Ergosterol Incidence

Since we detected ergosterol in non-inoculated trees which suggests the presence of endophytic fungi in the jack pine phloem, we compared ergosterol incidence among all three fungal species to demonstrate the close association of all three symbiotic fungi with the ergosterol. There were significant differences in ergosterol incidence between tissue types and among fungal species. Ergosterol incidence was higher in the lesions compared to non-infected phloem samples (*X*^2^_(1)_ = 77.94, *P* < 0.001). Over 77% of lesion samples and 17% of non-infected phloem samples contained detectable amounts of ergosterol. The incidence of ergosterol in lesion samples was significantly higher for each fungal treatment than their non-infected phloem samples (36 times greater for *L*. *longiclavatum*, 4 times greater for *G*. *clavigera*, and 2.7 times greater for *O*. *montium*). When we compared the ergosterol incidence between samples from mock-inoculated control trees and lesions, we found significantly higher ergosterol incidence in the former (*X*^2^_(1)_ = 27.64, *P* < 0.001). Over 77% of lesion samples and 27% of control samples from mock-inoculated control trees contained detectable amounts of ergosterol. Ergosterol incidence did not significantly differ between mock-inoculated control trees and non-infected phloem samples (*X*^2^_(1)_ = 1.51, *P* = 0.219).

### Ergosterol Concentration in Lesion Samples

**FIGURE 5:**
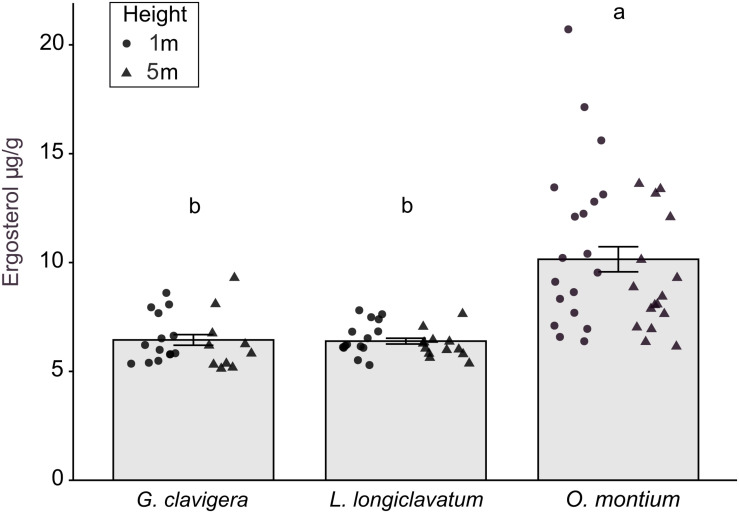
Ergosterol concentration (±SE) in lesion samples from fungal treatments in *Pinus banksiana* trees. Ergosterol concentration (μg g^– 1^) in lesions from trees inoculated with *Grosmannia clavigera*, *Leptographium longiclavatum* and *Ophiostoma montium* (*n* = 14, 15, and 19 trees, respectively). Ergosterol concentration did not vary between heights (1 and 5 m). Samples from the different heights were pooled together but the distribution of data was provided for each sample height. Different letters indicate that means are significantly different among treatments.

There was no significant difference in ergosterol concentrations between the 1 m and 5 m heights (*X*^2^_(1)_ = 0.34, *P* = 0.560) or any interaction effect between the fungal treatments and tree heights (*X*^2^_(2)_ = 0.60, *P* = 0.739). However, fungal inoculations significantly altered ergosterol concentrations (Figure 5; *X*^2^_(2)_ = 57.11, *P* < 0.001). Ergosterol concentrations were the highest in lesions collected from trees inoculated with *O*. *montium*, compared to those collected from trees inoculated with *G*. *clavigera* or *L*. *longiclavatum* (1.6 times higher in *O*. *montium* lesion compared to concentrations in lesions of either two fungi). Ergosterol concentration in lesions did not differ between the *G*. *clavigera* and *L*. *longiclavatum* treatments.

## Discussion

This is the first study to report the interspecific differences in ergosterol concentrations in lesions of three bark beetle-associated fungi infected jack pine trees. Ergosterol concentrations on inoculated jack pine phloem differed by fungal species. Of the three fungi examined, *O. montium* had higher ergosterol concentrations relative to *G*. *clavigera* and *L*. *longiclavatum*.

Ergosterol concentration is commonly used as a proxy to determine fungal biomass in a substrate (Xue et al., 2006; Porep et al., 2014). While we also detected low amounts of ergosterol in non-infected phloem samples and mock-inoculated control trees, which suggests the presence of fungal endophytes (Ganley et al., 2004; Giordano et al., 2009), the higher incidence and greater concentrations of ergosterol in lesions indicate differences in fungal growth and hyphal density among three fungal species tested. We show that while *O. montium* spreads slower than *G. clavigera* and *L*. *longiclavatum* in jack pine phloem, the fungus appears to colonize the tissue more densely (i.e., higher hyphal density) than the other two fungi. Earlier studies obtained similar results in both jack pine and lodgepole pine trees (Lee et al., 2006b; Rice et al., 2007; Rice and Langor, 2008; Cale et al., 2019).

The smaller yet more densely colonized area by *O*. *montium* might be related to its limited tolerance to the high moisture content and low oxygen availability of the fresh phloem and sapwood as suggested by Scheffer (1986). Previously it was shown that *G. clavigera* and *L*. *longiclavatum* are more tolerant to such conditions than *O. montium*, allowing them to grow faster in the tree tissues (Solheim, 1995; Solheim and Krokene, 1998). Furthermore, *G. clavigera* and *L. longiclavatum* can have higher growth rates than *O. montium* at temperatures between 5°C and 15°C whereas *O. montium* grows faster between 25°C and 30°C (Rice et al., 2008; Bleiker and Six, 2009; Ojeda-Alayon et al., 2017). We suspect that the higher spread of *G. clavigera* and *L. longiclavatum* in the phloem was also favored in the current study because, when we conducted this study, summer temperatures were usually below 20 °C, rarely exceeding 25 °C. Alternatively, differences in the ecology of all three fungi can also explain the slower growth of *O. montium*. *Ophiostoma montium* is considered as either a non-pathogenic or only a weakly pathogenic whereas *G. clavigera* and *L*. *longiclavatum* are considered as solely pathogenic fungi (Six and Paine, 1998). In general, variation in these traits can affect ability of each fungal species to colonize host tissues.

We found ergosterol concentrations ranging from 5.13 μg g^–1^ to 20.7 μg g^–1^ in jack pine phloem. These amounts are quite low compared to what was reported in Bentz and Six (2006) in which the concentration of ergosterol in MPB-infested lodgepole pine phloem was 100 μg g^–1^. The differences in the results of this and the earlier study may be attributed to differences between host tree species in terms of their secondary chemical profiles, how fungi were introduced to trees (artificial inoculation vs. natural beetle colonization), and analytical methods undertaken to quantify the ergosterol concentrations. First, although these two tree species share many secondary compounds, their concentrations show large variation, which may differentially affect fungal growth (Cale et al., 2017; Erbilgin, 2019; Wang et al., 2020). Second, in the current study, we quantified ergosterol concentration from individual fungal species, while the earlier study quantified ergosterol from trees colonized by MPB without assessing the relative concentrations from each fungus. Finally, the extraction efficiency and analytical sensitivity to quantify ergosterol could have differed between the methods used in the current study (GC-MS) and the previous study (high performance liquid chromatography, HPLC). Even though we initially used HPLC for ergosterol analysis, we were not satisfied with the analysis as we frequently found another unidentified sterol compound that co-occurred along with ergosterol almost at the same retention time and our further attempts did not allow us to separate the two compounds.

Mountain pine beetle populations contain two or more species of fungi across their range although the proportional abundance of each fungus shows variation. Earlier studies reported that some of the most common fungal pairings are *O*. *montium-G*. *clavigera*, followed by *O*. *montium-L*. *longiclavatum*, and the rarest being *G*. *clavigera-L*. *longiclavatum* in Alberta (Rice and Langor, 2009; Roe et al., 2011a). The prevalence of the different pairings may be related to temperature, which is commonly accepted as the mechanism driving the differential growth of fungi within MPB galleries (Six and Bentz, 2007; Rice et al., 2008; Ojeda-Alayon et al., 2017). *Grosmannia clavigera* and *L. longiclavatum* are more cold tolerant than *O. montium* (Six and Bentz, 2007; Rice et al., 2008). Hence, it is thought that the common association of *O*. *montium-G*. *clavigera* or *O*. *montium-L*. *longiclavatum* with MPB may minimize competition between fungi over the season as well as provide nutritional advantage to the beetle to persist in a wide range of environmental conditions (Six and Bentz, 2007; Rice et al., 2008).

Similarly, percent nitrogen concentration from infected tissues of jack pine varied by fungal species. Percent nitrogen was lower in the lesions of *G*. *clavigera* and *L*. *longiclavatum* than in those of *O*. *montium* and had an inverse relationship with the lengths and areas of lesions (i.e., longer or larger lesions do not necessarily imply higher nitrogen concentration). We propose that initially fungi likely remobilize nitrogen from the phloem to support their growth in the sapwood as nitrogen content can be 100 times higher in the phloem than the xylem (Mattson, 1980). This may also explain the larger lesion areas in the sapwood of trees inoculated with *G*. *clavigera* and *L*. *longiclavatum* than in those inoculated with *O*. *montium*. We suspect that faster growing fungi likely remobilized a higher rate of nitrogen from phloem to the xylem, resulting in a lower nitrogen concentration in the phloem, such as we observed in the lesions of *G*. *clavigera* and *L*. *longiclavatum*. Furthermore, as the fungal infection extends toward the xylem, the flow of nitrogen appears to shift its direction as reported nitrogen translocation from deep sapwood to the phloem. Although the current study focuses on nitrogen levels at different temporal phases of fungal infection of host trees, they all demonstrate that nitrogen movement between different tissues can be facilitated by symbiotic fungi and that flow of nitrogen changes as host colonization progresses. In general, our nitrogen results from jack pine tend to disagree with studies conducted in the lodgepole pine system. For instance, Goodsman et al. (2012) found no difference in nitrogen concentration among all three species of fungal symbionts of MPB, while Cook et al. (2010) found that *O. montium* is a less efficient nutrient concentrator than *G. clavigera in-vitro*. We suspect that differences between the current and earlier studies may be attributed to the time when nitrogen was measured as all these three studies differ when samples were collected from trees as well as a relatively lower percent nitrogen concentration of jack pine phloem than lodgepole pine phloem (0.32 % in jack pine vs. 0.51% in lodgepole pine from Cook et al. (2010) and 0.42% in lodgepole pine from Goodsman et al. (2012). Further research is needed to determine whether fungi associated with MPB can in fact concentrate nitrogen in jack pine phloem and the mechanisms involved with nitrogen depletion in early stages of the infection in the tree.

## Conclusion

In conclusion, *O*. *montium* differs from *G*. *clavigera* and *L*. *longiclavatum* in terms of acquiring nitrogen from plant tissues and producing ergosterol. It appears that these differences are linked to how fast each fungus grows as the growth rate of *O*. *montium* was slower from the other two fungi tested. Although our study provides the first information about relative concentrations of nitrogen and ergosterol from three species of fungi associated with MPB, considering the complexity of associations of beetles with a diversity of organisms including bacteria, mites, and nematodes, additional studies are needed to determine how the results of this study fit in the overall bark beetle biology. Furthermore, we only tested a single isolate (genotype) of each fungus even though bark beetle galleries likely contain multiple fungal genotypes and thus we need additional studies to determine the intraspecific variation of ergosterol and nitrogen among different genotypes of the fungi tested in this study. Likewise, the current study speculated a possible nitrogen translocation from the phloem to sapwood in live trees for the first six weeks of fungal inoculations. However, Elser has reported that translocation of nitrogen shifts directions from sapwood to phloem, suggesting that the pattern of nitrogen translocation may differ for the later stages of bark beetle development. Considering that the bark beetles feed on fungal infected tissues from early larval development stages until the emergence from the parental host as an adult, future studies should evaluate the nutritional role of each fungal symbiont in different developmental stages of MPB larvae until they complete their development. Finally, simultaneous inoculations with multiple species of fungi (i.e., *G*. *clavigera* and *O*. *montium*) in tree phloem may provide valuable information about how fungal competition affects ergosterol production or nitrogen concentration when two or more fungi co-occur.

## Data Availability Statement

The raw data supporting the conclusions of this article will be made available by the authors, without undue reservation.

## Author Contributions

SG-R, NE, and JC conceived the idea. SG-R and NE designed the methodology. AH, SG-R, and NE collected the samples in field. SG-R extracted and ran the samples in the GC/MS. JK and RR provided technical support for GC/MS analysis and troubleshooting. SG-R analyzed the data and wrote the manuscript. All authors contributed to subsequent revisions.

## Conflict of Interest

The authors declare that the research was conducted in the absence of any commercial or financial relationships that could be construed as a potential conflict of interest.

## Footnotes

1 http://nral.ualberta.ca
